# Supplementary material for: A Likelihood Approach for Real-Time Calibration of Stochastic Compartmental Epidemic Models
Source: PLoS Comput Biol. 2017 Jan 17;13(1):e1005257. doi: 10.1371/journal.pcbi.1005257 (PMC5240920; doi:10.1371/journal.pcbi.1005257)
Supplement: S1 File — (TAR.GZ) [file pcbi.1005257.s014.tar.gz › HSPH_Online-SI-Revision/EnKF07/n100-mild/n100-mild_EnKF_table0.pdf]

1	R0	{1.24125, 3.38982}	{2.21241, 7.49}	{{0.598473, 2.38404}, {3., 11.0577}}	{91.9615, 135.767}	inf	1822.	3073.93	{94.2415, 1217.68}	172.051	0.56385	newly	3621.	2173.36	{74., 1974.}	113.759	0.920781	Reff	0.993708	0.462684	{0.056781, 1.05733}	57.8306	ar	34 926.	177 243.	{165 490., 186 404.}	407.482
2	R0	{1.29554, 4.51878}	{2.15372, 9.99986}	{{0.00758213, 4.22844}, {3., 14.8505}}	{111.897, 123.87}	inf	2837.	1672.66	{215.327, 1339.53}	80.4758	0.536437	newly	4247.	1364.92	{172., 1719.}	89.1349	0.887126	Reff	0.958635	0.531513	{0.00344683, 0.999737}	51.7035	ar	42 828.	170 721.	{164 136., 179 400.}	298.62
3	R0	{1.2443, 3.64292}	{2.79744, 4.33325}	{{2.27796, 0.66786}, {3., 6.24744}}	{124.821, 53.2512}	inf	2191.	5225.62	{2589.15, 4256.79}	150.945	0.246052	newly	4114.	8421.28	{1961., 6947.}	163.248	0.329052	Reff	0.95617	0.635892	{0.341674, 0.833383}	49.4435	ar	37 087.	185 811.	{169 782., 230 157.}	401.013
4	R0	{1.38465, 3.43331}	{2.02534, 7.95641}	{{0.131676, 2.3343}, {3., 12.1225}}	{96.7352, 148.183}	inf	3745.	4254.32	{635.974, 6091.49}	112.229	0.482294	newly	6729.	3154.35	{415., 6031.}	83.7493	0.743979	Reff	0.908206	0.45152	{0.0258017, 0.69954}	51.3467	ar	49 511.	178 243.	{167 066., 214 394.}	260.007
5	R0	{1.25409, 4.1068}	{2.77342, 4.36981}	{{2.27545, 0.738558}, {3., 6.23499}}	{121.15, 45.4167}	inf	2471.	4162.65	{970.338, 4335.53}	86.3069	0.20118	newly	3936.	6696.58	{1393., 7264.}	129.613	0.311977	Reff	0.961511	0.637123	{0.343446, 0.934865}	46.3176	ar	38 571.	182 778.	{168 694., 191 012.}	373.874
6	R0	{1.28821, 4.2016}	{2.32224, 5.78444}	{{0.833959, 0.158129}, {3., 10.9945}}	{100.678, 114.007}	inf	2769.	3828.63	{154.641, 2880.08}	149.179	0.557465	newly	4394.	5761.44	{83., 22 414.}	151.569	0.802789	Reff	0.936864	0.536017	{0.216448, 1.09055}	50.0587	ar	41 468.	176 204.	{161 735., 188 254.}	324.915
7	R0	{1.24369, 2.56007}	{2.45418, 12.2662}	{{0.783444, 4.69343}, {3., 17.5076}}	{107.871, 379.135}	inf	2411.	3097.92	{850.607, 3251.25}	59.1457	0.192611	newly	6137.	2828.33	{338., 2523.}	96.4489	0.77259	Reff	0.952269	0.487354	{0.0685147, 0.773482}	60.2442	ar	38 603.	175 462.	{166 128., 190 136.}	354.53
8	R0	{1.232, 3.53942}	{2.07738, 10.0849}	{{0.150715, 4.25435}, {3., 14.9723}}	{119.221, 184.93}	inf	1816.	1612.85	{361.335, 1234.04}	76.7076	0.368247	newly	3274.	1216.21	{270., 1010.}	89.9325	0.797322	Reff	0.934361	0.496337	{0.0431532, 0.760276}	53.987	ar	34 555.	167 309.	{161 255., 171 734.}	384.182
9	R0	{1.36262, 3.85631}	{2.58351, 5.793}	{{1.55668, 3.58103}, {3., 11.7881}}	{89.599, 57.0742}	inf	3694.	6675.67	{387.017, 5319.33}	144.554	0.420095	newly	6191.	5857.92	{364., 6789.}	94.5899	0.562445	Reff	0.926772	0.593111	{0.323891, 0.700117}	53.3239	ar	48 410.	181 456.	{163 008., 228 223.}	274.832
10	R0	{1.28702, 4.67968}	{2.64602, 13.358}	{{2.15329, 3.8941}, {3., 19.626}}	{105.592, 194.582}	inf	2625.	1785.59	{76.3938, 4650.14}	106.988	0.839269	newly	3765.	1481.5	{33., 3908.}	97.0507	1.16413	Reff	0.941571	0.933297	{0.35287, 1.91803}	58.3236	ar	41 092.	186 742.	{164 705., 234 705.}	354.448
11	R0	{1.35752, 3.47129}	{2.73905, 4.26496}	{{2.28051, 0.620553}, {3., 6.3407}}	{101.768, 65.4069}	inf	3108.	4218.09	{474.744, 5717.08}	82.2111	0.255341	newly	6127.	7110.83	{1674., 8335.}	113.96	0.429714	Reff	1.02022	0.610602	{0.24571, 0.879971}	54.7501	ar	44 669.	183 355.	{167 941., 192 159.}	310.474
12	R0	{1.35929, 5.83647}	{2.6271, 13.3419}	{{2.15783, 3.92955}, {3., 19.5854}}	{93.2703, 146.685}	inf	3507.	1451.21	{212.623, 1869.51}	87.2977	0.760477	newly	4123.	1126.81	{135., 1837.}	90.156	1.04506	Reff	0.975236	0.90892	{0.29489, 2.15781}	54.2641	ar	46 715.	181 746.	{162 165., 246 266.}	289.053
13	R0	{1.28019, 3.29908}	{2.38919, 6.64434}	{{0.515352, 1.01814}, {3., 19.0334}}	{112.862, 166.314}	inf	2522.	8815.56	{1354.41, 7050.}	265.322	0.432049	newly	5146.	11 220.8	{1330., 14 931.}	164.277	0.379694	Reff	0.960053	0.55833	{0.0611153, 0.847608}	51.5003	ar	40 941.	184 918.	{165 049., 222 778.}	351.669
14	R0	{1.36661, 3.48318}	{2.43049, 11.2706}	{{0.360769, 4.53033}, {3., 16.8153}}	{97.4794, 223.573}	inf	3348.	2663.43	{306.43, 5325.6}	82.4333	0.539013	newly	5669.	2936.27	{132., 3028.}	104.718	0.952677	Reff	0.881791	0.553255	{0.165578, 0.642076}	57.4105	ar	48 672.	175 803.	{164 588., 180 690.}	261.2
15	R0	{1.35865, 3.84123}	{2.73819, 4.36092}	{{2.269, 0.569792}, {3., 6.37255}}	{101.538, 55.0052}	inf	3969.	4913.73	{2045.35, 6864.39}	73.5849	0.320519	newly	6860.	8038.93	{2081., 10 000.}	105.834	0.459656	Reff	0.953229	0.6355	{0.343483, 0.823968}	49.4877	ar	48 598.	182 276.	{166 720., 189 110.}	275.068
16	R0	{1.22879, 3.98329}	{2.63656, 13.4355}	{{2.17798, 4.03358}, {3., 19.6726}}	{114.566, 237.297}	inf	1615.	1749.55	{191.383, 1242.88}	95.4991	0.44579	newly	2786.	1172.88	{267., 1246.}	93.6461	0.849555	Reff	0.98678	0.989296	{0.470047, 2.17796}	48.6733	ar	34 405.	184 880.	{164 097., 248 095.}	437.363
17	R0	{1.34925, 3.64524}	{2.60555, 5.65888}	{{1.57583, 3.55342}, {3., 11.9244}}	{93.1113, 57.7591}	inf	3518.	6853.34	{616.059, 5469.32}	162.525	0.403185	newly	6327.	6399.44	{532., 12 175.}	98.6954	0.533349	Reff	0.979043	0.623624	{0.328209, 0.749871}	54.0883	ar	46 871.	182 671.	{163 482., 241 495.}	289.731
18	R0	{1.33054, 2.25275}	{2.31657, 3.94638}	{{1.6599, 0.333945}, {3., 7.02487}}	{81.4398, 130.547}	inf	3041.	4594.73	{836.298, 10 548.3}	137.494	0.419117	newly	8646.	5982.48	{861., 13 767.}	105.17	0.7428	Reff	0.969272	0.374565	{0.0677122, 0.671886}	65.3872	ar	44 336.	180 294.	{166 764., 215 772.}	308.622
19	R0	{1.28394, 3.69859}	{1.97249, 9.95505}	{{0.180112, 4.22669}, {3., 14.8166}}	{110.125, 169.158}	inf	2442.	1727.46	{663.803, 1458.41}	74.7391	0.381269	newly	4445.	1522.52	{330., 1533.}	89.6189	0.824547	Reff	0.980378	0.460883	{0.0397926, 1.11711}	62.4404	ar	39 558.	172 634.	{165 512., 187 465.}	336.406
20	R0	{1.2883, 2.26493}	{2.20441, 3.96574}	{{1.67843, 0.625789}, {3., 7.11286}}	{82.8424, 123.37}	inf	2636.	3429.72	{87.322, 3234.69}	118.822	0.517166	newly	7800.	4412.9	{110., 7585.}	98.7741	0.871027	Reff	1.01925	0.367251	{0.0734995, 0.700204}	67.1907	ar	40 768.	179 778.	{163 235., 225 855.}	341.463
21	R0	{1.34098, 2.78621}	{2.3197, 4.03499}	{{1.66857, 0.481691}, {3., 6.89275}}	{78.9457, 95.3653}	inf	3142.	3372.84	{42.394, 5819.41}	110.551	0.652884	newly	7245.	4363.72	{64., 6687.}	98.1761	0.901955	Reff	0.972707	0.364688	{0.0838559, 0.654809}	66.2535	ar	44 605.	179 645.	{164 646., 239 188.}	303.835
22	R0	{1.33783, 5.95911}	{2.6435, 12.9625}	{{2.13675, 3.95539}, {3., 19.6977}}	{97.5963, 138.145}	inf	3350.	1761.47	{70.0308, 1619.64}	95.0531	0.84258	newly	3826.	1443.04	{37., 1069.}	99.2143	1.09579	Reff	0.988844	0.885582	{0.330379, 2.10037}	57.9142	ar	45 418.	182 287.	{162 281., 244 502.}	301.353
23	R0	{1.33359, 3.3069}	{2.33693, 6.89854}	{{0.505384, 1.06685}, {3., 19.1442}}	{104.989, 170.471}	inf	3385.	9247.9	{4326.65, 7653.44}	179.651	0.334764	newly	6752.	11 982.8	{1656., 16 342.}	124.257	0.359043	Reff	0.971427	0.553781	{0.0786219, 0.794654}	51.6878	ar	46 245.	184 495.	{165 313., 223 577.}	298.951
24	R0	{1.30588, 2.56123}	{2.157, 6.23954}	{{0.948154, 3.41083}, {3., 9.55387}}	{85.7604, 144.9}	inf	2762.	1801.78	{300.472, 1394.5}	88.0322	0.579794	newly	6606.	1701.92	{221., 1762.}	89.8365	1.00246	Reff	0.932189	0.34216	{0.110403, 0.46525}	67.7878	ar	42 383.	174 910.	{165 650., 191 822.}	312.688
25	R0	{1.29813, 4.51849}	{2.67138, 13.4661}	{{2.17097, 3.9847}, {3., 19.5721}}	{105.787, 204.392}	inf	3099.	1589.6	{157.644, 1254.45}	97.6202	0.776705	newly	4627.	939.56	{54., 1073.}	90.2239	1.14889	Reff	0.990123	0.958502	{0.44511, 2.17095}	48.6016	ar	43 371.	183 300.	{163 889., 248 541.}	322.632
26	R0	{1.33231, 3.99953}	{2.24859, 7.30719}	{{0.590042, 2.29577}, {3., 11.0211}}	{86.4405, 104.803}	inf	3222.	3274.22	{153.533, 1682.52}	132.69	0.657373	newly	5352.	2355.04	{123., 2066.}	102.99	0.934596	Reff	0.96366	0.472874	{0.134643, 0.842583}	53.9813	ar	44 733.	173 744.	{161 703., 181 337.}	288.403
27	R0	{1.30555, 4.61445}	{2.67129, 13.427}	{{2.16368, 3.98103}, {3., 19.6086}}	{104.611, 198.634}	inf	3092.	1596.98	{277.438, 1357.81}	96.5872	0.752833	newly	4581.	957.82	{92., 919.}	89.7752	1.11749	Reff	0.983993	0.993913	{0.540588, 2.16366}	49.5125	ar	42 624.	184 672.	{164 337., 248 684.}	333.258
28	R0	{1.26819, 5.34969}	{2.0354, 9.72259}	{{0.125, 4.25454}, {3., 14.8744}}	{114.381, 89.9323}	inf	2467.	1627.31	{404.404, 1344.78}	76.6645	0.466346	newly	3182.	1336.77	{275., 1109.}	90.1556	0.774694	Reff	0.987438	0.515627	{0.0243163, 0.90987}	54.6564	ar	38 946.	170 419.	{164 267., 175 519.}	337.579
29	R0	{1.19925, 3.00394}	{2.71614, 6.25248}	{{1.81213, 3.14594}, {3., 8.43645}}	{126.487, 108.143}	inf	1551.	2781.79	{105.914, 1747.5}	153.482	0.424134	newly	3362.	3668.58	{74., 2544.}	137.842	0.749774	Reff	0.975746	0.516749	{0.0602238, 0.871576}	53.8026	ar	31 727.	177 215.	{167 610., 174 682.}	458.562
30	R0	{1.35749, 3.75483}	{2.40424, 6.80337}	{{0.484933, 1.12426}, {3., 18.9753}}	{104.055, 153.768}	inf	3853.	9406.16	{2646.18, 10 878.1}	154.877	0.301377	newly	6557.	12 388.3	{2023., 21 275.}	131.097	0.336431	Reff	0.934306	0.597396	{0.075699, 1.01362}	45.1453	ar	48 244.	186 499.	{166 175., 223 125.}	286.574
31	R0	{1.27131, 4.2161}	{2.27047, 7.28763}	{{0.483859, 2.38298}, {3., 11.0326}}	{92.7418, 95.4265}	inf	2579.	2569.11	{106.973, 1228.68}	125.123	0.630821	newly	4228.	1888.77	{125., 2058.}	101.915	0.910336	Reff	0.992664	0.457716	{0.0579484, 0.799653}	57.718	ar	38 299.	172 709.	{162 748., 183 756.}	350.949
32	R0	{1.31827, 6.18119}	{2.63774, 13.2285}	{{2.15347, 3.95711}, {3., 19.6201}}	{100.091, 134.705}	inf	3314.	1108.6	{171.054, 1084.8}	86.1606	0.837238	newly	3614.	846.03	{76., 1331.}	87.0362	1.0829	Reff	0.960834	0.927623	{0.152609, 2.15345}	60.0028	ar	45 262.	185 964.	{165 234., 249 583.}	310.861
33	R0	{1.20398, 1.35167}	{2.25221, 3.99987}	{{1.67443, 0.487064}, {3., 7.16088}}	{97.4323, 236.59}	inf	1581.	3217.13	{52.1522, 2934.46}	148.87	0.400971	newly	7272.	4073.36	{77., 7380.}	100.225	0.875066	Reff	0.95218	0.357594	{0.0703793, 0.704142}	66.3417	ar	33 329.	178 004.	{163 355., 225 807.}	434.299
34	R0	{1.32776, 3.146}	{2.58378, 5.71658}	{{1.55919, 3.58384}, {3., 11.7728}}	{94.5969, 81.7094}	inf	3143.	6937.26	{652.442, 5566.83}	168.134	0.347857	newly	6043.	6305.47	{627., 7638.}	93.3733	0.459886	Reff	0.90054	0.62713	{0.337547, 0.741386}	48.7923	ar	44 872.	181 368.	{162 889., 224 665.}	304.19
35	R0	{1.279, 1.11336}	{2.19824, 3.54384}	{{1.62565, 0.046004}, {3., 6.91534}}	{84.4653, 285.462}	inf	1968.	4218.32	{541.489, 3886.16}	162.682	0.28681	newly	7436.	6856.36	{358., 9263.}	123.636	0.711559	Reff	0.863977	0.397863	{0.037391, 0.834564}	65.8555	ar	41 033.	177 789.	{159 533., 219 038.}	333.282
36	R0	{1.20029, 1.94717}	{2.31827, 4.11213}	{{1.71521, 0.749705}, {3., 7.13304}}	{99.4886, 149.57}	inf	1687.	4327.14	{62.5015, 13 322.5}	204.231	0.500169	newly	5772.	5619.26	{86., 15 781.}	124.464	0.843617	Reff	0.983182	0.387594	{0.0777656, 0.753052}	64.4733	ar	33 234.	180 990.	{163 468., 243 987.}	444.592
37	R0	{1.33216, 3.69685}	{2.6536, 6.27316}	{{1.75761, 3.24183}, {3., 8.54827}}	{99.1956, 75.1326}	inf	3070.	3119.67	{357.161, 4979.96}	103.472	0.495972	newly	5464.	4340.3	{232., 5857.}	117.228	0.797097	Reff	0.949151	0.46447	{0.114771, 0.856836}	59.1227	ar	45 235.	176 140.	{163 812., 185 791.}	289.389
38	R0	{1.25434, 2.04314}	{2.48298, 4.05739}	{{1.97852, 0.33512}, {3., 7.09776}}	{100.664, 151.142}	inf	2420.	3003.5	{180.937, 2606.86}	128.59	0.583853	newly	7800.	5317.1	{128., 12 554.}	111.628	0.951584	Reff	0.972916	0.565727	{0.140349, 1.56289}	67.0656	ar	38 662.	183 379.	{165 670., 244 234.}	374.314
39	R0	{1.27466, 3.0741}	{2.47091, 11.4702}	{{0.575888, 4.66711}, {3., 17.1044}}	{108.521, 273.124}	inf	2301.	2924.09	{424.986, 3515.07}	67.7914	0.2698	newly	4560.	2858.1	{238., 2579.}	101.657	0.726446	Reff	0.912975	0.543907	{0.0567701, 0.810125}	58.6821	ar	40 181.	176 291.	{165 090., 180 552.}	338.742
40	R0	{1.33045, 2.68154}	{2.17827, 6.27877}	{{0.913357, 3.37664}, {3., 9.63282}}	{85.9439, 135.646}	inf	3224.	1535.97	{177.055, 1260.27}	83.8264	0.62747	newly	6981.	1375.25	{159., 1492.}	89.4721	1.02341	Reff	0.885574	0.336944	{0.113499, 0.461988}	66.9644	ar	45 951.	166 166.	{159 650., 185 294.}	261.617
41	R0	{1.37003, 2.83984}	{2.31224, 3.89592}	{{1.65565, 0.311507}, {3., 6.92433}}	{76.5573, 95.2223}	inf	4061.	3876.72	{855.543, 9598.66}	103.23	0.494665	newly	8819.	5009.37	{830., 10 430.}	93.7005	0.730348	Reff	0.953239	0.360896	{0.0702817, 0.634571}	66.0907	ar	48 926.	180 121.	{167 539., 243 225.}	270.254
42	R0	{1.3003, 2.94885}	{2.43212, 12.0257}	{{0.610432, 4.60846}, {3., 17.234}}	{102.091, 307.811}	inf	3021.	2705.07	{111.411, 4824.23}	83.0273	0.553666	newly	6589.	2729.7	{45., 3203.}	101.241	1.07555	Reff	0.931561	0.500528	{0.130857, 0.629362}	56.1113	ar	44 237.	173 185.	{163 904., 183 456.}	291.493
43	R0	{1.33756, 2.92653}	{2.32053, 4.08787}	{{1.66738, 0.453202}, {3., 7.02468}}	{80.7216, 91.4766}	inf	3675.	4071.02	{939.335, 11 470.4}	111.381	0.478819	newly	8332.	5241.42	{824., 12 659.}	96.8651	0.731384	Reff	0.990757	0.363062	{0.0734826, 0.653144}	66.9147	ar	47 342.	176 902.	{164 533., 227 460.}	276.331
44	R0	{1.35801, 2.92276}	{2.41491, 6.07635}	{{1.51258, 3.11547}, {3., 9.43107}}	{77.8274, 108.377}	inf	4090.	2097.22	{295.684, 2293.64}	86.0577	0.676211	newly	8641.	3271.07	{235., 1864.}	101.99	1.04713	Reff	0.933387	0.417258	{0.0727126, 0.564578}	64.1319	ar	47 940.	175 768.	{167 200., 184 410.}	267.678
45	R0	{1.31342, 4.02649}	{2.39064, 6.8101}	{{0.521737, 1.03686}, {3., 18.9019}}	{108.487, 145.021}	inf	3015.	9055.39	{2380.91, 7156.07}	211.791	0.377074	newly	4999.	11 948.5	{1322., 16 331.}	184.583	0.43437	Reff	0.979207	0.564438	{0.0627372, 0.815246}	51.8868	ar	42 995.	185 783.	{165 228., 223 213.}	332.105
46	R0	{1.37919, 3.75889}	{2.59652, 5.57483}	{{1.57833, 3.54801}, {3., 12.029}}	{88.2639, 54.1451}	inf	3990.	7569.27	{811.044, 7694.13}	165.473	0.43539	newly	7235.	7808.26	{515., 16 722.}	114.247	0.601479	Reff	1.00539	0.659207	{0.329891, 0.891223}	52.5659	ar	49 157.	185 205.	{164 600., 263 813.}	276.762
47	R0	{1.37458, 3.56117}	{2.58096, 5.54965}	{{1.5631, 3.57058}, {3., 11.914}}	{87.7636, 55.8378}	inf	4168.	7446.59	{326.434, 6426.45}	160.551	0.506413	newly	7293.	6877.54	{313., 18 567.}	101.693	0.659691	Reff	0.937725	0.639736	{0.336196, 0.779553}	51.7585	ar	48 478.	184 619.	{162 959., 230 073.}	280.831
48	R0	{1.33393, 3.78296}	{1.96886, 10.1266}	{{0.172732, 4.17625}, {3., 14.7374}}	{104.725, 167.691}	inf	3354.	1226.73	{320.233, 1349.29}	74.4143	0.587081	newly	5927.	923.25	{113., 1237.}	88.5512	1.03958	Reff	0.973742	0.413107	{0.0247051, 0.967224}	59.1101	ar	44 885.	165 575.	{161 374., 172 917.}	268.888
49	R0	{1.24918, 2.39046}	{2.44713, 12.2713}	{{0.7813, 4.73157}, {3., 17.6141}}	{106.523, 413.345}	inf	1875.	3242.96	{1353.82, 3514.23}	89.7442	0.207589	newly	4978.	2894.72	{613., 2725.}	100.246	0.691929	Reff	0.958921	0.508629	{0.0606306, 0.796614}	58.0012	ar	35 969.	176 073.	{166 099., 185 456.}	389.513
50	R0	{1.28281, 2.3032}	{2.14583, 8.44858}	{{0.194146, 2.37176}, {3., 12.5405}}	{108.295, 266.832}	inf	2262.	3505.58	{371.733, 4120.29}	138.361	0.453197	newly	6004.	2629.59	{176., 5695.}	88.0185	0.846467	Reff	0.938273	0.443397	{0.0362892, 0.653924}	53.5074	ar	40 223.	173 225.	{163 798., 190 668.}	330.661
